# Editorial: Emergency radiology: between unsolved problems and new challenges

**DOI:** 10.3389/fradi.2026.1798556

**Published:** 2026-02-25

**Authors:** Graziella Di Grezia, Mariano Scaglione, Stefania Tamburrini, Giacomo Sica

**Affiliations:** 1 Department of Life Sciences, Health, and Healthcare Professions, Link Campus University, Rome, Italy; 2 Radiology Department of Surgery, Medicine and Pharmacy, University of Sassari, Sassari, Italy; 3 Radiology Unit, ASL Napoli 1 Centro, Naples, Italy; 4 Radiology Unit, Ospedale Sacro Cuore di Gesu—Fatebenefratelli, Benevento, Italy

**Keywords:** active bleeding imaging, computed tomography, emergency radiology, non operative management, spleen

## 1 Introduction

Emergency radiology is undergoing a profound transformation, suspended between persistent clinical challenges and new opportunities driven by rapidly evolving technologies. Increasing case complexity, pressure on decision-making times, and the growing need for sustainable diagnostic pathways demand a re-evaluation of traditional models of emergency care.

The Research Topic “*Emergency Radiology: Between Unsolved Problems and New Challenges*” was conceived with this awareness: to explore how imaging is no longer merely a diagnostic tool but a strategic component capable of shaping the entire patient pathway.

**Figure 1:**
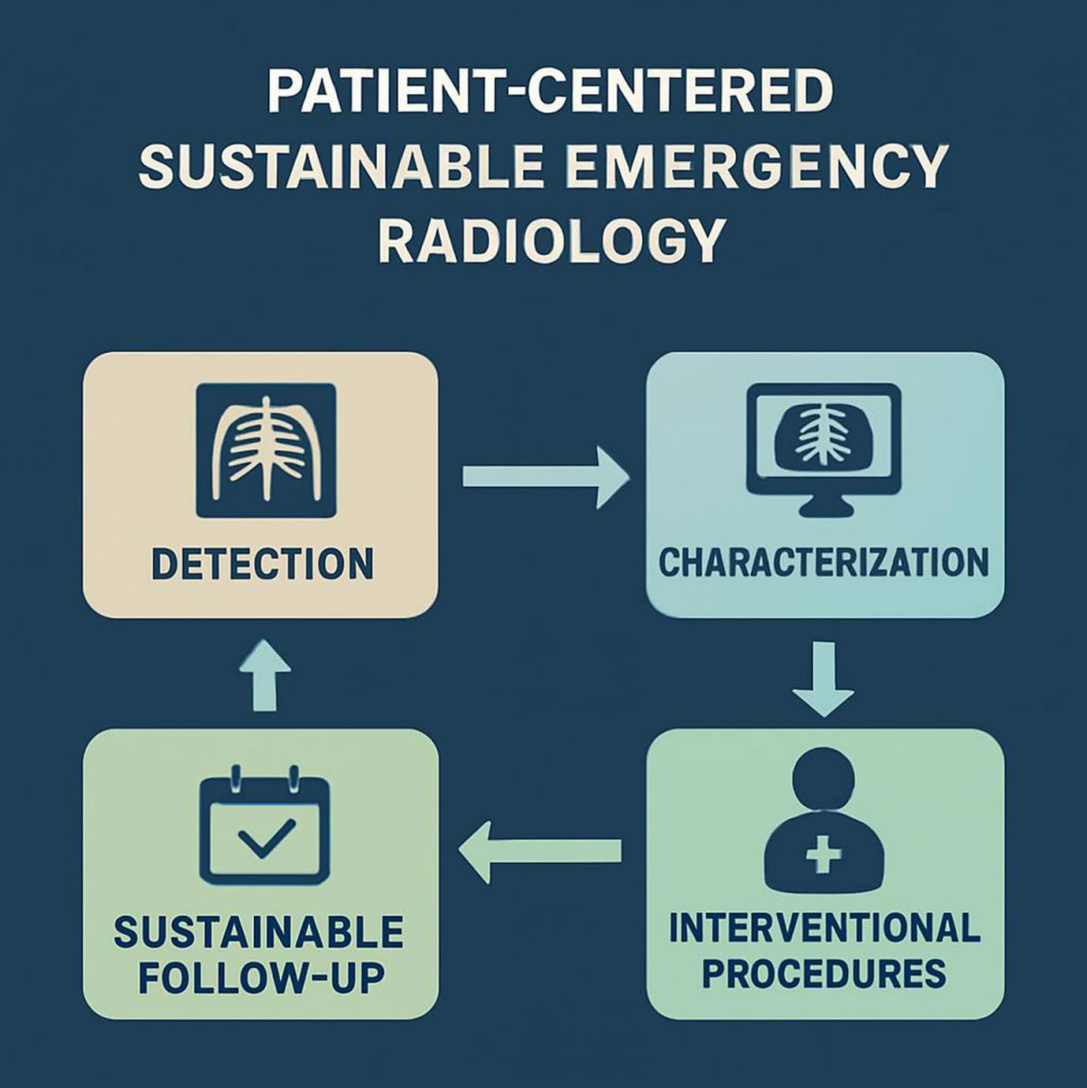
Conceptual workflow of patient-centered sustainable emergency radiology.

The contributions collected in this Special Issue converge on a unifying principle: emergency radiology must not only respond to emergencies—it must anticipate them (Figure 1).

## 2 Detection: identifying acute and rapidly evolving conditions

Several contributions highlight the central role of imaging in the early detection of rare or rapidly progressive emergencies.

The case of spontaneous splenic rupture reported by Romano et al. illustrates the diagnostic challenge posed by conditions that mimic more common presentations. Contrast-enhanced CT remains essential for distinguishing traumatic from non-traumatic etiologies and guiding the choice between conservative and surgical management (1–5).

Similarly, acute portal vein thrombosis in non-cirrhotic patients, described by Zhou et al., demonstrates how timely CT evaluation enables rapid diagnosis and stratification of therapeutic options. In both scenarios, imaging functions as the first decisive step in recognizing high-risk conditions.

## 3 Characterization: defining the clinical scenario with precision

Once detected, acute conditions require accurate characterization to guide management.

Weng et al. describe pediatric duodenal intramural hematoma, a condition in which multimodal imaging—CT for rapid assessment, MRI for detailed evaluation, and ultrasound for monitoring—allows a comprehensive understanding of lesion extent and evolution.

Karimialavijeh et al. address acute myocarditis, emphasizing the value of cardiac MRI in tissue characterization. Their exploration of low-field MRI systems highlights how technological innovation can expand access to advanced diagnostic tools, even in emergency settings.

## 4 Interventional procedures: imaging-guided therapeutic decision-making

Imaging does not merely diagnose; it increasingly directs treatment.

In portal vein thrombosis, imaging findings determine whether patients are candidates for systemic thrombolysis or require more complex interventional approaches.

Usai et al. extend this concept beyond the emergency domain, presenting a hybrid model that integrates interventional radiology, functional imaging, and robotic surgery. Selective prostatic artery embolization with ICG–Lipiodol, followed by fluorescence-guided lymphadenectomy, exemplifies how imaging can refine surgical precision and reduce unnecessary dissections. Although elective, this model anticipates future applications in acute care.

## 5 Sustainable follow-up: low-impact, accessible monitoring pathways

Sustainability emerges as a transversal value across all contributions.

In pediatrics, ultrasound provides a radiation-free tool for dynamic follow-up of duodenal hematoma, reducing the need for repeated CT examinations.

In cardiac imaging, AI-enhanced low-field MRI offers a promising avenue for accessible, repeatable follow-up of myocarditis, especially in resource-limited settings. These approaches demonstrate how imaging can support long-term patient management while minimizing exposure and optimizing resources.

## 6 Conclusions

Taken together, the contributions of this Research Topic outline a radiology that evolves along a coherent pathway: detection, characterization, intervention, and sustainable follow-up, all centered on the patient.

As Editors, we hope that this Research Topic will stimulate further collaborations, promote the dissemination of sustainable models, and contribute to building an emergency radiology that is more prepared, more accessible, and more innovative.
